# Bifurcation study, phase portraits and optical solitons of dual-mode resonant nonlinear Schrodinger dynamical equation with Kerr law non-linearity

**DOI:** 10.1016/j.heliyon.2024.e34416

**Published:** 2024-07-14

**Authors:** Yong Wu, Miguel Vivas-Cortez, Hamood Ur Rehman, El-Sayed M. Sherif, Abdul Rashid

**Affiliations:** a General Education Department, Anhui Xinhua University, Hefei, China; b School of Physical and Mathematical Sciences, Faculty of Exact and Natural Sciences, Pontifical Catholic University of Ecuador, Av. 12 October 1076. Section, Quito 17-01-2184, Ecuador; c Department of Mathematics, University of Okara, Okara, Pakistan; d Mechanical Engineering Department, College of Engineering, King Saud University, P.O. Box 800, Al-Riyadh 11421, Saudi Arabia

**Keywords:** Dual-mode resonant non-linear Schrodinger equation, Kerr law, Extended hyperbolic function method (EHFM), Soliton solutions, Bifurcation analysis

## Abstract

This study investigates the dynamic characteristics of the dual-mode resonant non-linear Schrodinger equation with a Bhom potential. Hydrodynamics, nonlinear optical fibre communication, elastic media, and plasma physics are just a few of the mathematical physics and engineering applications for this model. The study aims to achieve two main objectives: first, to discuss bifurcation analysis, and second, to extract optical soliton solutions using the extended hyperbolic function method. The study successfully derives various wave solutions, including bright, singular, periodic singular and dark solitons, based on the governing model. The findings conferred in this article show a crucial advancement in understanding the propagation of waves in non-linear media. Additionally, bifurcation of phase portraits of ordinary differential equation consistent with the partial differential equation under consideration is conducted. We also highlight specific constraint conditions that ensure the presence of these obtained solutions. The existing literature shows that these methods are first time applied on this model.

## 1 Introduction

Nonlinear partial differential equations (NLPDEs) form a fundamental framework renowned for their exceptional accuracy in modeling a diverse range of nonlinear physical phenomena [1], [2], [3], [4], [5], [6], [7], [8], [9]. These equations have become a central focus in modern nonlinear science, especially in the investigation of integrable systems. The study of NLPDEs holds significant value, offering profound insights into complex behaviors across various scientific fields. Key disciplines such as nonlinear optics and plasma physics rely heavily on NLPDEs for practical applications. These equations are important in describing a broad spectrum of phenomena. In the field of NLPDEs, a dedicated group of researchers has been actively engaged in both qualitative and quantitative analyses, with a particular emphasis on exploring soliton solutions in recent years [10], [11], [12], [13], [14], [15], [16], [17], [18], [19].

The nonlinear Schrödinger equation (NLSE) is a fascinating NLPDE with significant applications in many fields such as condensed matter physics, nonlinear optics and fluid mechanics [20], [21]. In the field of nonlinear optics, the NLSE is particularly important for modeling phenomena like four wave mixing and self phase modulation. The NLSE is specially intriguing because it can support soliton solution of NLPDEs. Two-mode or as it is seldom called dual-mode type equations have newly tempted significantly more research in the non-linear sciences. Since two-mode equations in the conventional framework investigate the improvisational wave interactions. Wazwaz [22] gained two-mode forth order Burgers and multiple kink solutions for the dual-mode Sharma-Tassa-Olver equation with the help of simplified Hirota's method. Biswas et al. [23] gained two-mode optical solitons for their model with the help of tanh-coth expansion method. Seadawy et al. [24] gained dual wave soliton solutions for two-mode resonance non-linear Schrödinger equation (RNLSE) with the help of exp(−*ϕ*)-expansion method and Zayed et al. [25] gained many classes of Jacobi elliptic solutions, singular, bright and dark solitons with the help of new extended auxiliary equation method, tanh-coth method and the unified Riccati equation method. There are several different works allied to the two-mode Korteweg-de Vries (KdV) equation [26], [27], [28], [29], [30], [31], [32], [33]. Dual-mode form is a new class of non-linear partial differential equations (PDEs) which has the following form [34], [35].

(1) $$u_{tt}-s^{2}u_{xx}+(D_{t}-\alpha sD_{x})N(u,u_{x},...)+(D_{t}-\beta sD_{x})L(u_{kx})=0.$$

Where $D_{t}=\frac{\partial }{\partial t}$, $D_{x}=\frac{\partial }{\partial x}$, $N(u,u_{x},...)$ is the non-linear term, $L(u_{kx})$; k≥2 is the linear term involved in the equation and u(x,t) is the unknown field function, s≥0 represents the phase velocity, β≤±1 represents the dispersion parameter, α≤±1 represents the parameter of non-linearity. If we put s=0 and integrate Eq. (1) with respect to time, the two-mode equation is reduced to first order partial differential equation in time t. Applications of the non-linear Schrödinger's equations cited in physics, quantum mechanics [36], [37], [38] and other related new non-linear physics [39], [40], [41]. Furthermore, Alquran [42], considered the brunt of phase velocity on the two-mode wave solutions for Schrödinger's equation integrating distinct modes of non-linearities. The incentive in back of this effort is to investigate dual waves also their distribution under the impact of embedded phase velocity parameter for RNLSE. It shows a very key role in Modelung fluids [43]. The general form of the RNLSE given below:

(2) $$iu_{t}+\frac{1}{2}u_{xx}+S(|u{|}^{2})u+\sigma (\frac{|u{|}_{xx}}{\left|u\right|})u=0$$

where u and S respectively represent the complex envelope function and generalized version of nonlinearity. The generalized RNLSE takes the form

(3) $$i(u_{tt}-s^{2}u_{xx})+(D_{t}-\alpha sD_{x})(S(|u{|}^{2})u+\sigma (\frac{|u{|}_{xx}}{\left|u\right|})u)+(D_{t}-\beta sD_{x})(\frac{1}{2}u_{xx})=0.$$

In this work, we study the generalized RNLSE with Kerr law [44], [45], [46] given as

(4) $$i(u_{tt}-s^{2}(u_{xx}))+(D_{t}-\alpha sD_{x})((|u{|}^{2})u)+\sigma (\frac{|u{|}_{xx}}{\left|u\right|})u)+(D_{t}-\beta sD_{x})(\frac{1}{2}u_{xx})=0.$$

The core of this work is to investigate dynamics of two-mode RNLSE using Kerr law. We also analyzed the bifurcations and dynamics behavior for our model. Furthermore, under parametric conditions, important solutions such as bright solutions, bright singular solutions, periodic singular solutions and dark solutions were observed by using the extended tanh function method [47]. The motivation for proposing the EHFM and bifurcation analysis stems from their novelty and effectiveness. The effectiveness of our proposed method is supported by existing literature, which demonstrates its successful application in solving numerous equations. Specifically, in our EHFM, we utilize two distinct forms to extract solutions, including bright, dark, periodic-singular, and singular solutions. [55], [56], [57], [58]. By identifying critical points and examining stability, bifurcation analysis efficiently identifies qualitative changes in system behavior as parameter changes [52], [53], [54]. This is the first time these methods have been applied in this context. They offer a straightforward approach to solve the complex equations, making them particularly suitable for our research objective.

**Figure 1:**
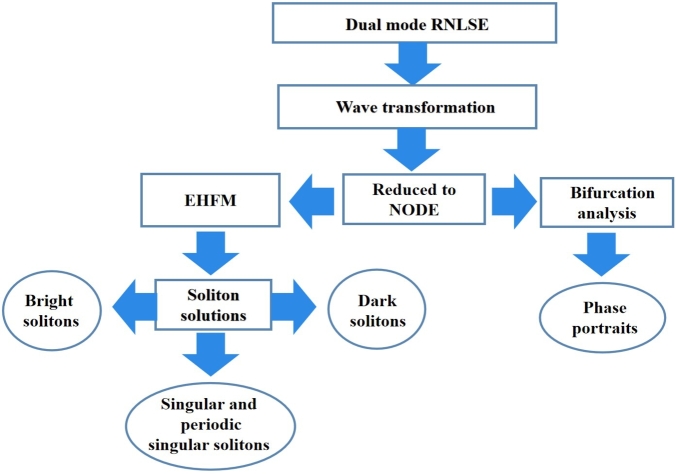
Flow chart diagram.

The paper is categorized as: In Section 2, we presented related works. Section 3 contains materials and methods which is categorized as extended hyperbolic function method (EHFM) and bifurcation analysis. Results are discussed in Section 4. Section 5 contains discussions. In Section 6 we discussed about conclusions while Section 7 contains future work. We also added the flow chart diagram in Fig. 1.

## 2 Related works

In various research investigations, a variety of techniques have been investigated to solve the (RNLSE) such as the new extended direct algebraic technique [49] has been utilized in the axial direction, resulting in the plotting of optical soliton solutions including dark, bright, and dark-bright solitons. Additionally, the modified auxiliary equation method [50] has been applied, incorporating the Kerr law, parabolic law, and anti-cubic law of nonlinearities, leading to the extraction of novel soliton solutions such as dark, periodic, singular and bell-shaped solitons. Furthermore, the modified extended tanh-function method [51] has been employed, revealing various types of solutions including Weierstrass elliptic doubly periodic solutions, Jacobi elliptic functions solutions, triangular solutions, hyperbolic solutions, bright and dark solitons, exponential solutions, and hyperbolic solutions. By introducing EHFM and bifurcation analysis in this context, we contribute to expanding the toolkit of mathematical techniques available for solving complex equations.

## 3 Materials and methods

### 3.1 Extended hyperbolic function method

The extended hyperbolic function approach for getting numerous exact solutions of particular Schrödinger equation is presented here. Let us consider the non-linear partial differential equation

(5) $$P(u,u_{t},u_{x},u_{tt},u_{xx},u_{tx},...)=0.$$

Let the wave traveling equations [48]

(6) $$u(x,t)=\Gamma (\xi )e^{\iota \lambda (x+\omega t)},\;\xi =x-ct.$$

Γ(ξ) is a real valued function λ,ω and *c* are constants. Re-writing Eq. (6) in Eq. (5), we attain the non-linear ODE as follows:

(7) $$Q(\Gamma ,\Gamma_{\xi },\Gamma_{\xi \xi },\Gamma_{\xi \xi \xi },...)=0.$$

The solutions for the Eq. (7) represented as:

**Form-I**:

As solutions of the soliton are generally sech*ξ* polynomials. Let the solution of non-linear ordinary differential equation (ODE) Eq. (7) has the structure

(8) $$\Gamma (\xi )=\sum_{j=0}^{N}a_{j}\phi^{j}(\xi ),\;a_{N}\neq 0,$$

Where N is the balancing number and $c_{j}$ (j=1,2,...N) are real coefficients to be determined. Let ϕ(ξ) satisfy the non-linear ODE Eq. (7) is of the form.

(9) $$\phi^{\prime }(\xi )=\frac{d\phi }{d\xi }=\phi \sqrt{m+n\phi^{2}},\;m,n\in R.$$

If m>0 and n>0, then

(10) $$\phi_{1}(\xi )=-\sqrt{\frac{m}{n}}\text{csch}[\sqrt{m}(\xi +\xi_{0})].$$

If m<0 and n>0, then

(11) $$\phi_{2}(\xi )=\sqrt{\frac{-m}{n}}\text{sec}[\sqrt{-m}(\xi +\xi_{0})].$$

If m>0 and n<0, then

(12) $$\phi_{3}(\xi )=\sqrt{\frac{m}{-n}}\text{sech}[\sqrt{m}(\xi +\xi_{0})].$$

If m<0 and n>0, then

(13) $$\phi_{4}(\xi )=\sqrt{\frac{-m}{n}}\text{csc}[\sqrt{-m}(\xi +\xi_{0})].$$

If m<0 and n>0, then

(14) $$\phi_{5}(\xi )=\text{cos}[\sqrt{-m}(\xi +\xi_{0})]+i\text{sin}[\sqrt{-m}(\xi +\xi_{0})].$$

If m=0 and n>0, then

(15) $$\phi_{6}(\xi )=\pm \frac{1}{\sqrt{n}(\xi +\xi_{0})}.$$

If m=0 and n<0, then

(16) $$\phi_{7}(\xi )=\pm \frac{1}{\sqrt{-n}(\xi +\xi_{0})}.$$

**Form-II**:

As solutions of the soliton generally are also tanh*ξ* polynomials. Let the solution of nonlinear ODE Eq. (7) has the structure as in Eq. (8). Let ϕ(ξ) satisfy the non-linear ODE Eq. (7) is of the form.

(17) $$\phi^{\prime }(\xi )=\frac{d\phi }{d\xi }=m+n\phi^{2},\;m,n\in R.$$

If mn>0, then

(18) $$\phi_{8}(\xi )=\text{sgn}(m)\sqrt{\frac{m}{n}}\text{tan}[\sqrt{\text{mn}}(\xi +\xi_{0})].$$

If mn>0, then

(19) $$\phi_{9}(\xi )=-\text{sgn}(m)\sqrt{\frac{m}{n}}\text{cot}[\sqrt{\text{mn}}(\xi +\xi_{0})].$$

If mn<0, then

(20) $$\phi_{10}(\xi )=\text{sgn}(m)\sqrt{-\frac{m}{n}}\text{tanh}[\sqrt{-\text{mn}}(\xi +\xi_{0})].$$

If mn<0, then

(21) $$\phi_{11}(\xi )=\text{sgn}(m)\sqrt{-\frac{m}{n}}\text{coth}[\sqrt{-\text{mn}}(\xi +\xi_{0})].$$

If m=0 and n>0, then

(22) $$\phi_{12}(\xi )=-\frac{1}{n(\xi +\xi_{0})}.$$

If m<0 and n=0, then

(23) $$\phi_{13}(\xi )=m(\xi +\xi_{0}).$$

Remember that, sgn represent the Sign function.

### 3.2 Solution of the equation (4)

Let us suppose the solution for traveling waves have the form

(24) $$u(x,t)=\Gamma (\xi )e^{\iota \lambda (x+\omega t)},\;\xi =x-ct.$$

Putting Eq. (24) into Eq. (4), we get a set of non-linear ODEs. After splitting into real parts and imaginary parts, we have:

(25) $$\lambda^{2}(2s^{2}-2\omega^{2}-\lambda \omega +\beta s\lambda )\Gamma +\lambda (2\omega -2\alpha s)\Gamma^{3}+(\lambda \omega -2s^{2}+2c^{2}-3\lambda \beta s-2\alpha s\sigma \lambda -2\lambda c+2\sigma \omega \lambda )\Gamma^{\prime \prime }=0.$$

(26) $$\lambda (\lambda c+4s^{2}-2\lambda \omega +3\lambda \beta s+4\omega c)\Gamma -(c+\alpha s)2\Gamma^{3}-(\beta s+c+2\sigma (\alpha s+c))\Gamma^{\prime \prime }=0.$$

**Form-I**:

Balancing principle determines N=1 for Eq. (26). Thus Eq. (8) gives

(27) $$\Gamma (\xi )=a_{0}+a_{1}\phi (\xi )$$

After putting Eq. (27) and Eq. (9) in Eq. (26) and equating coefficients for each power of ϕ(ξ) to zero, a system of different equations is obtained, which gives

(28) $$a_{0}=0,\beta =\pm 1,\alpha =\pm 1,c=\mp s,\lambda =\mp 2s.$$

Solutions of the non-linear ODE Eq. (26) are given below:

If m>0, n>0, then

(29) $$u_{1}(x,t)=-a_{1}\sqrt{\frac{m}{n}}\text{csch}[\sqrt{m}(\xi +\xi_{0})]e^{\text{i}\text{λ}(x+\omega t)}.$$

If m<0, n>0, then

(30) $$u_{2}(x,t)=a_{1}\sqrt{\frac{-m}{n}}\text{sec}[\sqrt{-m}(\xi +\xi_{0})]e^{\text{i}\text{λ}(x+\omega t)}.$$

If m>0, n<0, then

(31) $$u_{3}(x,t)=a_{1}\sqrt{\frac{m}{-n}}\text{sech}[\sqrt{m}(\xi +\xi_{0})]e^{\text{i}\text{λ}(x+\omega t)}.$$

If m<0, n>0, then

(32) $$u_{4}(x,t)=a_{1}\sqrt{\frac{-m}{n}}\text{csc}[\sqrt{-m}(\xi +\xi_{0})]e^{\text{i}\text{λ}(x+\omega t)}.$$

If m<0, n>0, then

(33) $$u_{5}(x,t)=a_{1}\text{cos}[\sqrt{-m}(\xi +\xi_{0})]+i\text{sin}[\sqrt{-m}(\xi +\xi_{0})]e^{\text{i}\text{λ}(x+\omega t)}.$$

If m=0, n>0, then

(34) $$u_{6}(x,t)=\frac{a_{1}}{\sqrt{n}(\xi +\xi_{0})}e^{\text{i}\text{λ}(x+\omega t)}.$$

If m=0, n<0, then

(35) $$u_{7}(x,t)=\frac{a_{1}}{\sqrt{-n}(\xi +\xi_{0})}e^{\text{i}\text{λ}(x+\omega t)}.$$

**Form-II**:

Balancing principle determines N=1 for Eq. (26). Thus Eq. (8) gives

(36) $$\Gamma (\xi )=a_{0}+a_{1}\phi (\xi ).$$

After putting Eq. (36) and Eq. (17) in Eq. (26) and equating coefficients for each power of ϕ(ξ) to zero, a system of different equations is obtained, which gives

(37) $$a_{0}=0,\beta =\pm 1,\alpha =\pm 1,c=\mp s,\lambda =\mp 2s.$$

If mn>0, then

(38) $$u_{8}(x,t)=a_{1}\text{sgn}(m)\sqrt{\frac{m}{n}}\text{tan}[\sqrt{\text{mn}}(\xi +\xi_{0})]e^{\text{i}\text{λ}(x+\omega t)}.$$

If mn>0, then

(39) $$u_{9}(x,t)=-a_{1}\text{sgn}(m)\sqrt{\frac{m}{n}}\text{cot}[\sqrt{\text{mn}}(\xi +\xi_{0})]e^{\text{i}\text{λ}(x+\omega t)}.$$

If mn<0, then

(40) $$u_{10}(x,t)=a_{1}\text{sgn}(m)\sqrt{-\frac{m}{n}}\text{tanh}[\sqrt{-\text{mn}}(\xi +\xi_{0})]e^{\text{i}\text{λ}(x+\omega t)}.$$

If mn<0, then

(41) $$u_{11}(x,t)=a_{1}\text{sgn}(m)\sqrt{-\frac{m}{n}}\text{coth}[\sqrt{-\text{mn}}(\xi +\xi_{0})]e^{\text{i}\text{λ}(x+\omega t)}.$$

If m=0, n>0, then

(42) $$u_{12}(x,t)=-\frac{a_{1}}{n(\xi +\xi_{0})}e^{\text{i}\text{λ}(x+\omega t)}.$$

If m<0, n=0, then

(43) $$u_{13}(x,t)=a_{1}m(\xi +\xi_{0})e^{\text{i}\text{λ}(x+\omega t)}.$$

### 3.3 Bifurcation solitons of equation (26)

In this section, we will study qualitative behavior of the governing model with the help of Galilean transformation [52], [53], [54]. Eq. (26) is transformed into planer dynamical system as;

(44) $$\begin{array}{cc} \frac{d\Gamma }{d\xi } & =u,\\ \frac{du}{d\xi } & =b_{1}\Gamma -b_{2}\Gamma^{3}, \end{array}$$

where $b_{1}$=$\frac{(4\omega c+4s^{2}+3\lambda \beta s-2\lambda \omega +\lambda c)\lambda }{c+\beta s+2\sigma (c+\alpha s)}$ and $b_{2}$=$\frac{2(c+\alpha s)}{c+\beta s+2\sigma (c+\alpha s)}$.

This is Hamiltonian system and has integral

(45) $$H(\Gamma ,u)=\frac{u^{2}}{2}-b_{1}\frac{\Gamma^{2}}{2}+b_{2}\frac{\Gamma^{4}}{4}=h,$$

where h is Hamiltonian constant. Firstly, note that system of Eqs. (44) have three equilibrium points which are:

(46) $$E_{1}=(0,0),E_{2}=(\sqrt{\frac{b_{1}}{b_{2}}},0),E_{3}=(-\sqrt{\frac{b_{1}}{b_{2}}},0).$$

Also, the Jacobian matrix for the system will be:

(47) $$J(\Gamma ,u)=\left(\begin{array}{cc} 0\; & 1\\ b_{1}-3b_{2}\Gamma^{2}\; & 0 \end{array}\right),$$

The determinant form of the Jacobi matrix is as follows:

(48) $$det[J(\Gamma ,u)]=(3b_{2}\Gamma^{2}-b_{1}).$$

Here (Γ,0) represents equilibrium points for the system of Eqs. (44). The equilibrium points can be categorized as saddle point if det[J(Γ,u)]<0, center point if det[J(Γ,u)]>0 or a cuspidal point if det[J(Γ,u)]=0. Different cases are possible for parameters $b_{1}$ and $b_{2}$.

**Case 1:** When $b_{1}>0$ and $b_{2}<0$.

The determinant for the Jacobi matrix is calculated for that case which is negative or less than 0. By choosing distinct values to the involving parameters ω=1.6,λ=−0.3,α=0.5,s=1,c=−1,β=2.5,σ=0.5, three critical points $E_{1}=(0,0),E_{2}=(\iota ,0),E_{3}=(-\iota ,0)$ have been obtained from the system of Eqs. (44).

**Figure 2:**
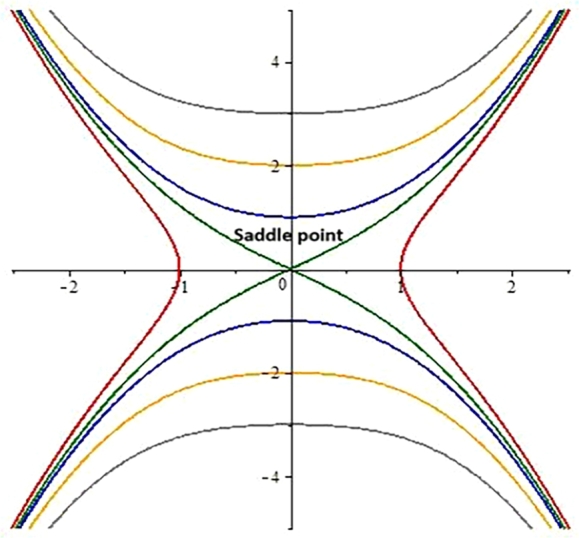
2D phase portrait analysis.

We get point $E_{1}$ being a real number as a saddle point as shown in Fig. 2.

**Case 2:** When $b_{1}<0$ and $b_{2}>0$.

The determinant for the Jacobi matrix is calculated for that case which is positive or greater than 0. By assigning different values to the involving parameters α=−0.5,s=1,c=1,β=−2,σ=2,λ=1,ω=−0.01, three equilibrium points $E_{1}=(0,0),E_{2}=(\iota ,0),E_{3}=(-\iota ,0)$ have been obtained from the system of Eqs. (44).

**Figure 3:**
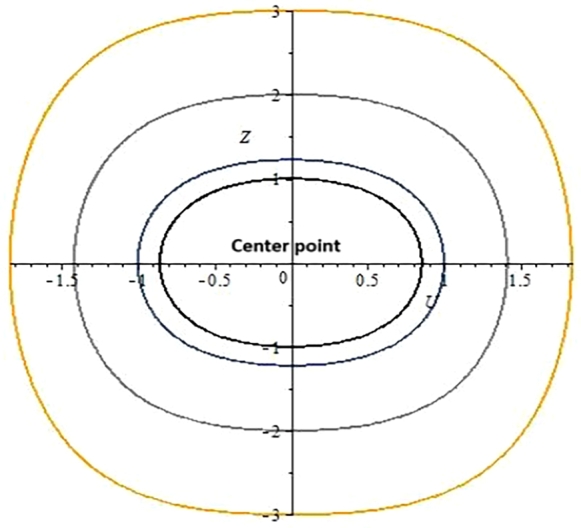
2D phase portrait analysis.

We get point $E_{1}$ being a real number as a center point as shown in Fig. 3.

**Case 3:** When $b_{1}<0$ and $b_{2}<0$.

The determinant for the Jacobi matrix is calculated for that case which is positive or greater than 0. By assigning different values to the involving parameters ω=1.92,λ=1,α=0.5,s=1,c=−1,β=2.5,σ=0.5, three points $E_{1}=(0,0),E_{2}=(1,0),E_{3}=(-1,0)$ have been obtained from the system of Eqs. (44).

**Figure 4:**
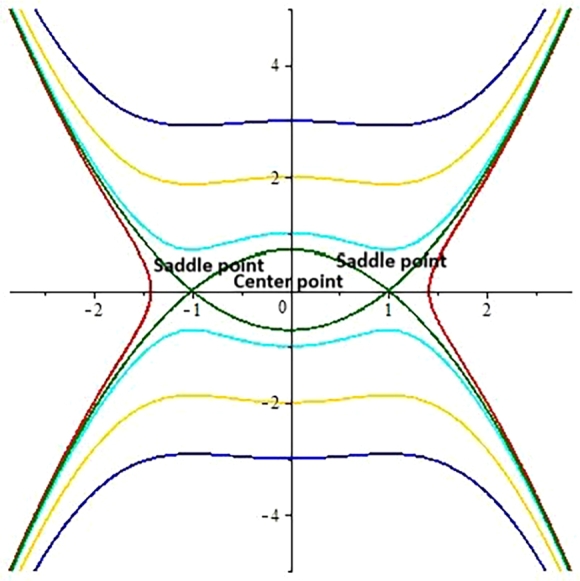
2D phase portrait analysis.

The critical points $E_{2}$ and $E_{3}$ represent saddle points and $E_{1}$ is center point as shown in Fig. 4.

**Case 4:** When $b_{1}>0$ and $b_{2}>0$.

The determinant for the Jacobi matrix is calculated for that case which is negative or less than 0. By assigning different values to the involving parameters α=−0.5, s=1, c=1, β=−2, σ=2, λ=1, ω=1, $\beta_{3}=4$, three critical points $E_{1}=(0,0)$, $E_{2}=(1,0)$, $E_{3}=(-1,0)$ have been obtained from the system of Eqs. (44).

**Figure 5:**
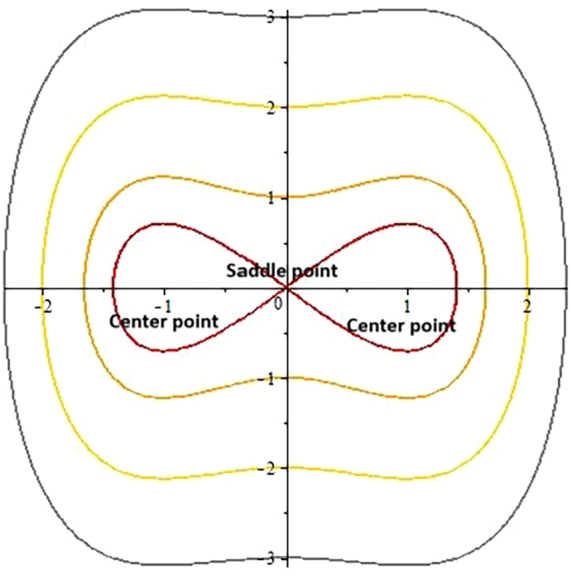
2D phase portrait analysis.

The points $E_{2}$ and $E_{3}$ represent center points while $E_{1}$ is a saddle point as shown in Fig. 5.

## 4 Results

**Figure 6:**
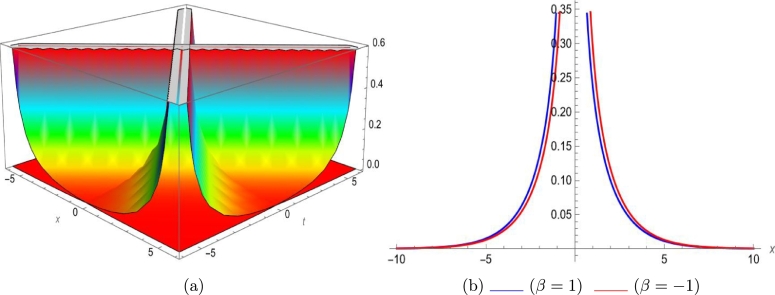
3D and 2D dual-mode bright soliton profile for *y*_1_(*x*,*t*).

**Figure 7:**
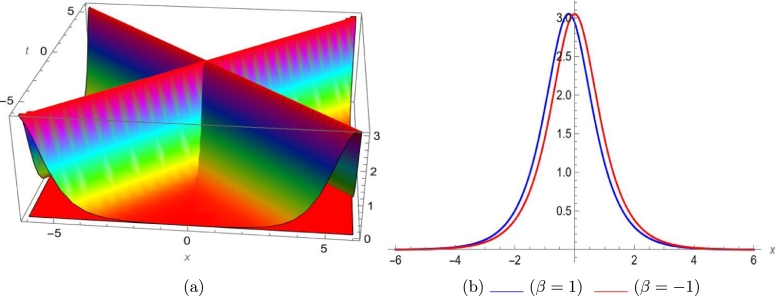
3D and 2D dual-mode singular soliton profile for *y*_3_(*x*,*t*).

**Figure 8:**
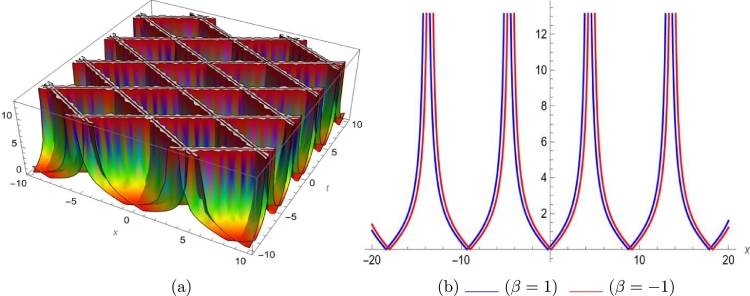
3D and 2D dual-mode periodic soliton profile for *y*_8_(*x*,*t*).

**Figure 9:**
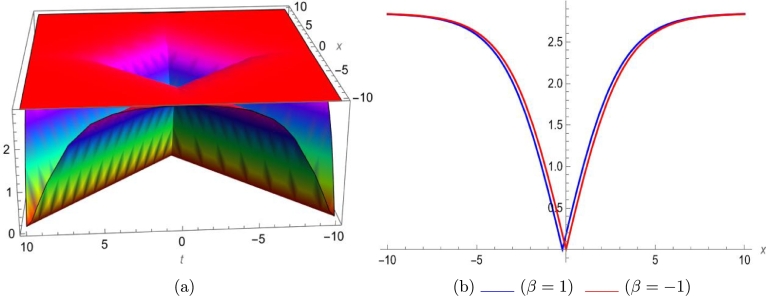
3D and 2D dual-mode dark soliton profile for *y*_10_(*x*,*t*).

**Figure 10:**
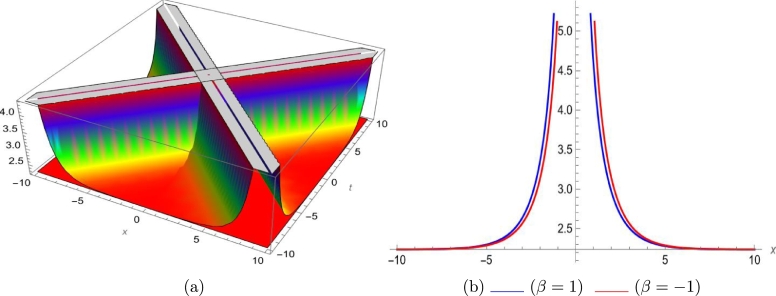
3D and 2D dual-mode singular soliton profile for *y*_11_(*x*,*t*).

Mathematical expressions of gained solutions for dual mode RNLSE are more useful by exploring the nature of soliton reliant on their graphical representations. The soliton identified in Eq. (29) represent dual wave singular soliton solution. Fig. 6 with assumed parameters m=0.5, n=0.1, s=1, $\xi_{0}=0.1$, ω=0.1, $a_{1}=0.1$, σ=0.1 shows modulus of $u_{1}(x,t)$ in 3*D* and 2*D*. The soliton identified in Eq. (31) represent dual wave bright soliton solution. Fig. 7 with assumed parameters m=1.9, n=−0.1, s=1, $\xi_{0}=0.1$, ω=0.1, $a_{1}=0.7$, σ=0.1 shows modulus of $u_{3}(x,t)$ in 3*D* and 2*D*. The soliton identified in Eq. (38) represent dual wave periodic soliton solution. Fig. 8 with assumed parameters σ=0.1, $\xi_{0}=0.1$, n=0.1, ω=0.5, s=1.9, $b_{1}=0.5$, m=1.2 shows modulus of $u_{8}(x,t)$ in 3*D* and 2*D*. The soliton identified in Eq. (40) represent dual wave dark soliton solution. Fig. 9 with assumed parameters σ=0.1, $\xi_{0}=0.1$, n=−0.1, ω=0.1, s=1, $b_{1}=0.9$, m=1 shows modulus of $u_{10}(x,t)$ in 3*D* and 2*D*. The solitons identified in Eq. (41) represent dual wave singular soliton solution. Fig. 10 with assumed parameters σ=0.1, $\xi_{0}=0.1$, n=−0.2, ω=0.1, s=1, $b_{1}=1,m=1$ shows modulus of $u_{11}(x,t)$ in 3*D* and 2*D*. Furthermore, we have carried out a qualitative behavior for our model by studying bifurcation. We transformed it into a planer dynamical system by using the Galilean transformation and execute a bifurcation analysis. Phase portraits for distinct parametric values have been shown in Figure 1, Figure 2, Figure 3, Figure 4. Bifurcation sets with equilibrium points, center points, saddle point have been evaluated and can be seen by phase portraits.

## 5 Discussions

These solitons have been obtained by using Mathematica. Obtained solutions constitute free parameters and autonomous capricious functions that support to interpret soliton solutions. We find bright, singular, dark, periodic singular soliton solutions. Where as bright soliton solutions can hold their form and stability across long distances, they are used in optical fibers for long-distance communication. Dark soliton solutions use in order to manipulate light pulses for information transmission, optical fibers for signal processing. They are also used in ultracold atomic gases to study quantum processes like quantum turbulence. Singular solitons are used in fluid dynamics to describe phenomena such as wave breaking and rogue waves in bodies of water. They are also used in nonlinear optics to create localized structures that may transmit information without dispersion. In order to effectively process signals and localize energy, periodic singular soliton solutions are useful for simulating wave propagation in nonlinear systems like periodic photonic lattices. This allows researchers to investigate phenomena like discrete breathers and soliton management. Here *β* which stands for the dispersive factor, shows that soliton changes its position by taking its values +1 and −1. The matured solutions for the soliton equations are momentous and unusual which were not fixed in literature. Some of the commonly found solutions in the literature are presented in Table 1. Phase portraits use a graphing technique to display the evolution of a system across time by comparing its variables. Their use in physics, engineering, biology, and other sciences involves understanding and controlling the motions and patterns of systems, and they also help in predicting the system's future behavior.

**Table 1:** Comparison between our solutions and existing papers.

Our solutions	Solutions from existing papers
If *θ*_1_ = *m*, *θ*_2_ = *n*,*u*_1_(*x*,*t*)=*u*_2,2_(*x*,*t*), then (29) becomes $u_{2,2}(x,t)=a_{1}\sqrt{\frac{\theta_{1}}{\theta_{2}}}\text{csch}[\sqrt{m}(\xi +\xi_{0})]e^{\text{i}\text{λ}(x+\omega t)}$	If *ν* = *ξ*_0_, *λ* = *ψ*, *ω* = *ϖ*, then solution (65) of [59] becomes then $u_{2,2}(x,t)=a_{1}\sqrt{\frac{\theta_{1}}{\theta_{2}}}\text{csch}[\sqrt{m}(\xi +\xi_{0})]e^{\text{i}\text{λ}(x+\omega t)}$.
If *θ*_1_ = *m*, *θ*_2_ = *n*,*u*_2_(*x*,*t*)=*u*_2,10_(*x*,*t*), then (30) becomes $u_{2,2}(x,t)=a_{1}\sqrt{\frac{\theta_{1}}{\theta_{2}}}\text{sec}[\sqrt{m}(\xi +\xi_{0})]e^{\text{i}\text{λ}(x+\omega t)}$	If *ν* = *ξ*_0_, *λ* = *ψ*, *ω* = *ϖ*, then solution (73) of [59] becomes then $u_{2,10}(x,t)=a_{1}\sqrt{\frac{\theta_{1}}{\theta_{2}}}\text{sec}[\sqrt{m}(\xi +\xi_{0})]e^{\text{i}\text{λ}(x+\omega t)}$
If *θ*_1_ = *m*, *θ*_2_ = *n*,*u*_3_(*x*,*t*)=*u*_2,1_(*x*,*t*), then (31) becomes $u_{2,1}(x,t)=a_{1}\sqrt{\frac{\theta_{1}}{\theta_{2}}}\text{sech}[\sqrt{m}(\xi +\xi_{0})]e^{\text{i}\text{λ}(x+\omega t)}$	If *ν* = *ξ*_0_, *λ* = *ψ*, *ω* = *ϖ*, then solution (64) of [59] becomes then $u_{2,1}(x,t)=a_{1}\sqrt{\frac{\theta_{1}}{\theta_{2}}}\text{sech}[\sqrt{m}(\xi +\xi_{0})]e^{\text{i}\text{λ}(x+\omega t)}$

## 6 Conclusions

This paper investigates a novel dual-mode RNLSE for Kerr non-linearity. The model illustrates the simultaneous movement of two distinct waves, and we successfully obtain dual-mode optical including bright, singular, periodic singular and dark soliton solutions using EHFM. The simultaneous propagation of these waves is effectively depicted in 3D and 2D plots in Figure 6, Figure 7, Figure 8, Figure 9, Figure 10. Moreover, bifurcation analysis of phase portraits of ordinary differential equations consistent with the partial differential equation under consideration has been conducted, contributing to a cavernous understanding for the system's behavior. The existing literature indicates that these methods are applied to this type of equation for the first time. Additionally, we have included a comparison in the discussion section to highlight the effectiveness and novelty of our approach. The rulings for the work can give a beneficial perception towards growth for data communication systems. The enhance in number of two-mode waves can sport the role of hauler wave for communication of data in distinct directions.

## 7 Future work

In the future, we plan to apply numerical methods to solve the proposed equation, which will provide an additional perspective on its behavior and characteristics. Furthermore, we intend to explore stochastic and fractional versions of the equation to broaden our understanding of its dynamics in different contexts. Additionally, we aim to extract various types of solutions such as multisoliton, breather and lump type soliton solutions to analyze the model's behavior. These extensions will contribute to a more comprehensive understanding of the equation and its potential applications across different disciplines.

## CRediT authorship contribution statement

**Yong Wu:** Writing – review & editing, Validation, Software, Formal analysis. **Miguel Vivas-Cortez:** Writing – review & editing, Visualization, Project administration, Funding acquisition, Formal analysis. **Hamood Ur Rehman:** Writing – review & editing, Visualization, Supervision, Investigation, Conceptualization. **El-Sayed M. Sherif:** Writing – review & editing, Resources, Formal analysis. **Abdul Rashid:** Writing – original draft, Software, Methodology.

## Declaration of Competing Interest

The authors declare that they have no known competing financial interests or personal relationships that could have appeared to influence the work reported in this paper.

## Data Availability

Data included in article/supp. material/referenced in article.
